# Supplementary material for: Plasma membrane flipping of Syntaxin-2 regulates its inhibitory action on insulin granule exocytosis
Source: Nat Commun. 2022 Oct 31;13:6512. doi: 10.1038/s41467-022-33986-3 (PMC9622911; doi:10.1038/s41467-022-33986-3)
Supplement: Supplementary file 1 — Supplementary information [file 41467_2022_33986_MOESM1_ESM.pdf]

Supplementary information

Plasma membrane flipping of Syntaxin-2 regulates its inhibitory action on insulin granule exocytosis

Fei Kang[1,2], Li Xie[1], Tairan Qin[1], Yifan Miao[1], Youhou Kang[1], Toshimasa Takahashi[1], Tao Liang[1,2], Huanli Xie[1], Herbert Y. Gaisano[1,2]**

[1]Department of Medicine, Temerty Faculty of Medicine, University of Toronto, Toronto, Ontario M5S 1A8, Canada.

[2]Toronto General Hospital Research Institute, University Health Network, 200 Elizabeth Street, Toronto, Ontario M5G 2C4, Canada.

***Correspondence to**: Herbert Y. Gaisano, M.D. or Fei Kang, Ph.D.; Email: herbert.gaisano@utoronto.ca or fei.kang@utoronto.ca

Supplementary Table 1. Information on human pancreatic islet donors.

No.	Age (yr)	Gender	BMI (kg/m^2)	HbA1c (%)	Isolation ID
1	60	male	21.4	5.6	R305
2	20	male	19.8	5.3	R308
3	31	female	30.3	5.0	R314
4	68	male	27.8	5.0	R319
5	24	female	31.6	4.5	R321
6	26	male	27.0	5.5	R326
7	48	male	22.7	5.2	R328
8	25	male	24.6	4.9	R335
9	36	male	23.3	5.3	R340
10	27	male	24.6	5.7	R346
11	75	male	30.2	5.2	R354
12	79	male	23.5	5.2	R355
13	45	female	29.7	5.1	R356
14	54	male	29.4	5.4	R362
15	66	male	25.6	4.9	R369
16	55	female	29.1	N/A	R371
17	47	male	32.9	N/A	R381
18	28	female	31.4	3.6	R395
19	35	female	37.2	5.7	R403
20	25	female	23.0	5.1	R443
21	33	male	24.2	5.6	R445

Supplementary Table 2. Information on human pancreas donors wherein we had prepared pancreatic slices. COD, cause of death.

No.	Age (yr)	Gender	BMI (kg/m^2)	COD	Source
1	77	female	22.9	other	Trillium Gift of Life Network, Canada, 2020
2	25	male	19.0	Anoxia	Trillium Gift of Life Network, Canada, 2020
3	61	female	29.5	stroke	Trillium Gift of Life Network, Canada, 2020
4	48	female	18.7	CVA due to closed head injury	Trillium Gift of Life Network, Canada, 2021
5	26	male	19.7	stroke	Trillium Gift of Life Network, Canada, 2021
6	20	male	17.1	Respiratory arrest	Trillium Gift of Life Network, Canada, 2021
7	19	male	27.0	ICH	Trillium Gift of Life Network, Canada, 2021
8	38	female	22.0	Anoxia	Trillium Gift of Life Network, Canada, 2021

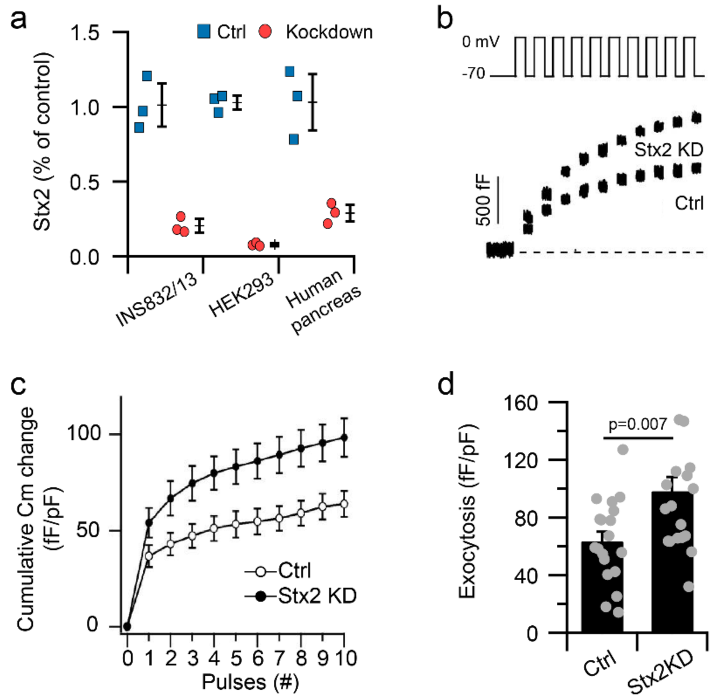

Supplementary Figure 1. Stx2 knockdown in human β-cells increases insulin secretion but does not affect calcium channel (Ca_v) activity.

(**a**) Quantification of Stx2 knockdown is shown. Values are mean ± S.D. of three independent experiments. (**b**) Cell membrane capacitance (Cm) changes in human islet β cells infected with Ad-control (Control) or Ad-shStx2 (Stx2 KD) viruses. Representative recordings of exocytosis during of a train of 500-ms depolarizations from -70 mV to 0 mV in human islet β cells. (**c**) The cumulative changes in cell membrane capacitance normalized to basal Cm values (fF/pF) in Control (n=20) and Stx2 KD (n=20) β-cells. Values represent the mean ± SEM. (**d**) The Cm changes from depolarization-induced insulin exocytosis (Control: 63.7 ± 6.6 fF/pF; Stx2 KD:98.3 ± 10 fF/pF). Values represent the mean ± SEM, n = 20, two-tailed unpaired Student's *t*-test.

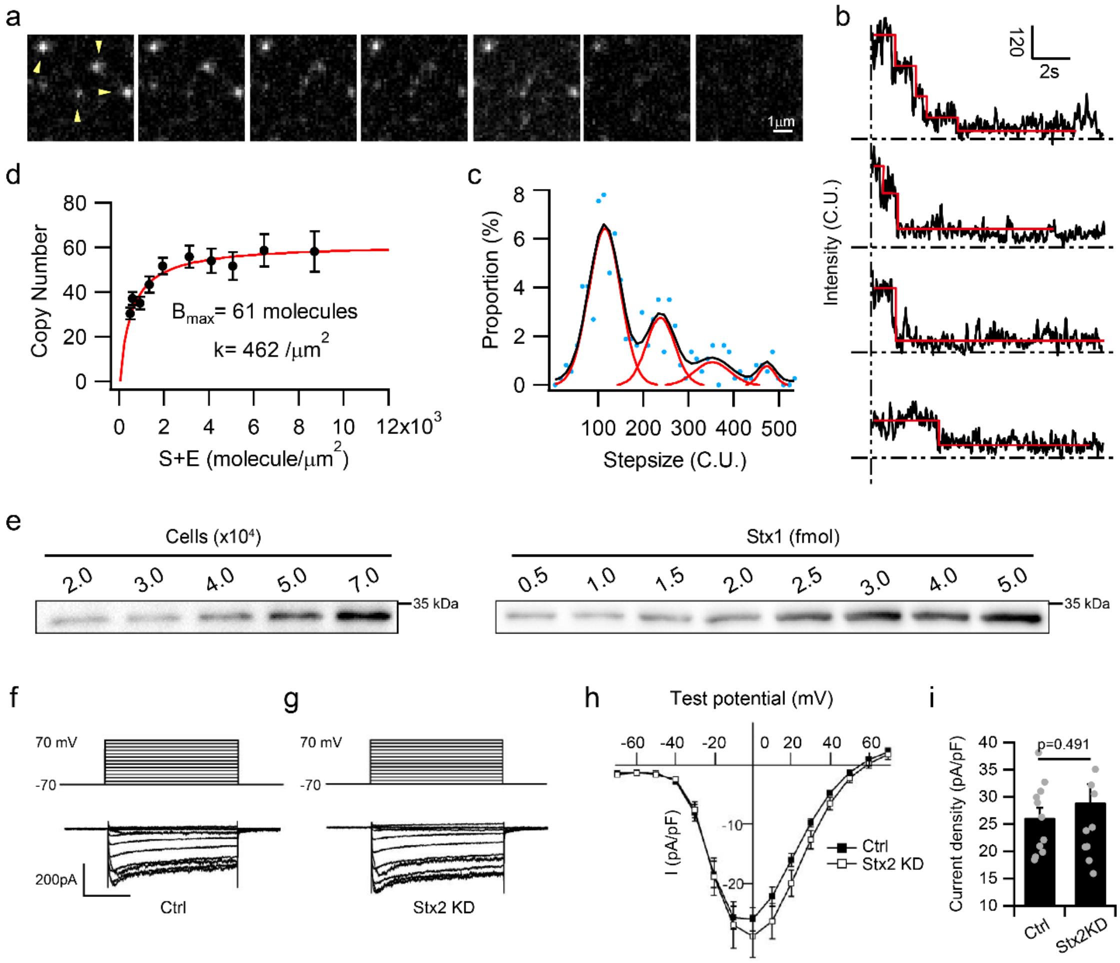

Supplementary Figure 2. Analysis of the stepwise bleaching behavior of Stx2-mScarlet.

(**a**) Representative images show mScarlet bleaching in fixed human β-cell coexpressing Stx2-mScarlet (n = 3 independent experiments). (**b**) Representative traces showing the bleaching steps of the puncta in (**a**). Red lines indicate visually identified bleaching steps. (**c**) Frequency distribution of the size of mScarlet bleaching steps. Dots denote frequency, the collective distribution fitted with multi-component Gaussian function, sum of each peak shown by the black line. (**d**) Relationship between granule-associated Stx1a and the global surrounding expression, S, in human β-cells. (**e**) Quantitative western blots determine the endogenous copy number of Stx1 by comparing purified recombinant Stx2 with human β-cell lysates. Human β-cells recognized by $HPi2^+/HPa3^-$ and counted by FACS (n = 3 independent experiments). We estimated that the overall mean copy number of Stx1 is 190±22 × 10^3/cell, that is, ~600 molecules/μm^2 (with an average cell-surface of 315 μm^2 for human β-cell [1]). The endogenous copy number of Stx1a, B_{endo} = 34 molecules (derived from 61*600 / (462 + 600) = 34.46) bound per cluster. (**f-g**) Voltage-gated Ca^{2+} currents in human islet β-cells were recorded after infection with Ad-control (Control) or Ad-Stx2 knockdown (Stx2 KD) viruses. Representative traces showing Ca_V currents recorded in the whole-cell mode from control and Stx2-KD human β-cells. Membrane potential was held at -

70 mV and depolarized for 300 ms from -70 to +70 mV in 10-mV increments. (**h**) Current-voltage relationship of Ca_Vs from control (n=12) and Stx2-KD (n=12) β-cells. Currents were normalized to cell capacitance to yield current density. Values are means ± SEM. (**i**) Bar chart showing maximum increase in current densities under stimulation of 0 mV voltage (n=12, from three independent experiments, values are mean + SEM, two-tailed unpaired Student's *t*-test).

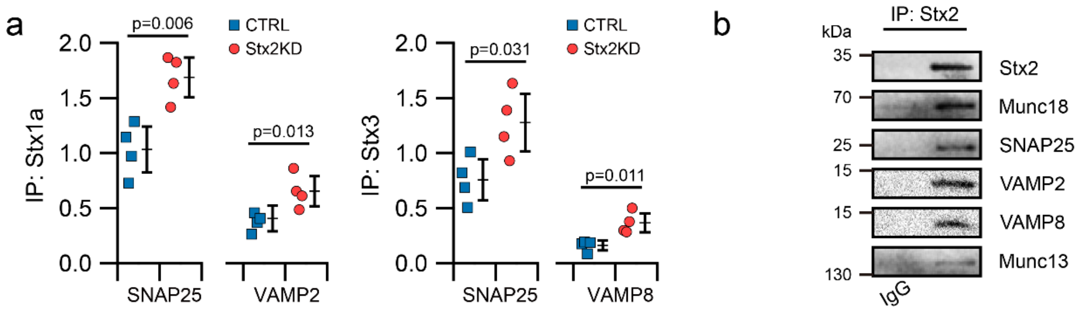

Supplementary Figure 3. Stx2 deletion enhances exocytosis by facilitating trans-SNARE complex assembly.

(**a**) Quantification of co-immunoprecipitated SNARE complexes corresponding to Fig. 3a. Values are mean ± S.D., two-tailed unpaired Student's *t*-test, from four independent experiments. (**b**) Exocytotic proteins immunoprecipitated with Stx2 from human islets extracts. Normal rabbit IgG was used as a control. This is representative of three experiments.

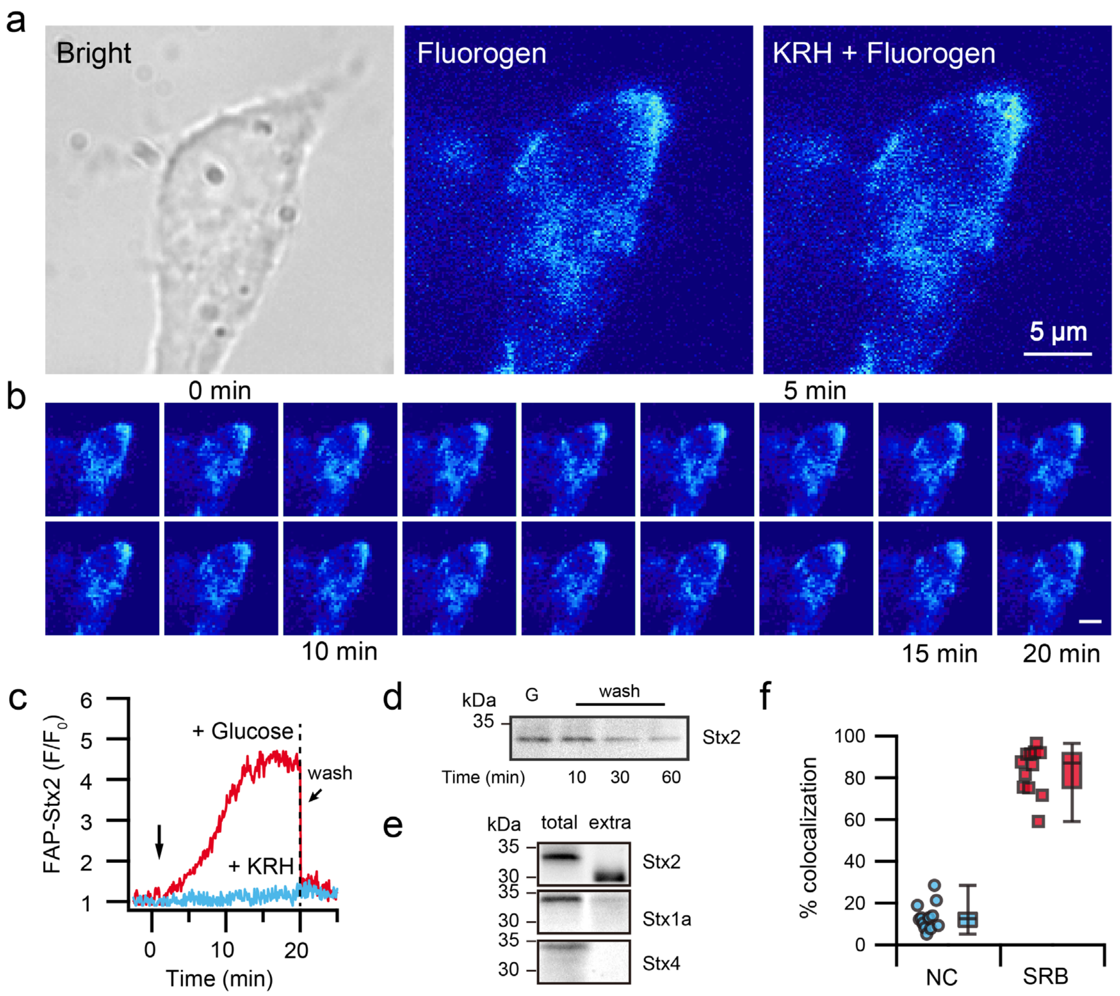

Supplementary Figure 4. Stx2 flipping characterization and colocalization analysis with exocytosis.

(**a**) INS-832/13 cell expressing FAP-Stx2 was stimulated with Krebs-Ringer HEPES (KRH) buffer without glucose as negative control for comparison to the glucose-induced flipping shown in Fig. 4b (n = 3 independent experiments). (**b**) Time lapse images show that no discernible changes of the global FAP-Stx2 observed in the cell in (**a**). Scale bar, 5 µm. (**c**) The time course of FAP-Stx2 flipping in the presence and absence of glucose demonstrated that glucose-induced Stx2 flipping occurs in less than 1 min, and reaches stable maximum after about 15-20 min. (**d**) Immunoblot evaluating extracellular Stx2 from concentrated supernatants following exposure to 5 µg/mL BoNT/C1-LC. INS-832/13 cells expressing FAP-Stx2 were incubated with glucose for 15 min and then washed with KRH buffer (n=3 independent experiments). (**e**) Stx2 is able to flip across the plasma membrane and be released into the extracellular space, which is not the case with Stx1a and Stx4. The indicated 'total' proteins were from total cell lysate and the indicated 'extra' samples are the accumulated extracellular supernatant during 30-min glucose stimulation and concentrated 10-fold after 1.5 mg/mL collagenase releasing treatment of intact islets (n = 3 independent experiments). (**f**) Colocalization analysis of exocytosis visualized by SRB with flipping events indicated by FAP-Stx2 in human pancreatic tissue slices comparing to negative control (NC) using a simulated

complete spatially random distribution. Maximum intensity projection of SRB events overlapped with FAP-Stx2 hotspots were calculated. Box: 25th and 75th percentiles. Line: median. Whiskers: smallest to the largest values. Markers: average of individual cell.

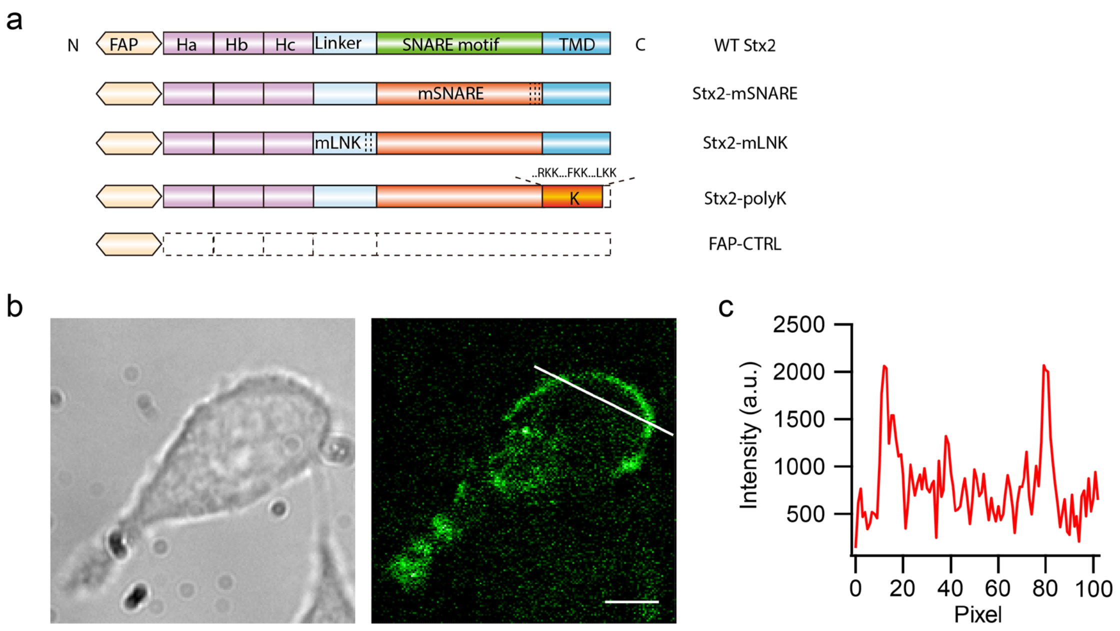

Supplementary Figure 5. Stx2 mutants used for the flipping analysis.
(**a**) Schematic representation of Stx2 mutants used in our study. FAP tag was seamlessly assembled at the N-terminal end of Stx2. To generate Stx2-mSNARE, the Lysine and Arginine of Stx2 adjacent to TMD (dashed line indicated) were mutated as negatively charged aspartic and glutamic acid. Stx2-mLNK introduced more positively charged amino acid in the linker region between Habc and mSNARE domain by inserting Lysine and Arginine. The plasma membrane-anchoring polyK region (SPGRK KFPLK IFKKP LKK) of STIM1 replaced the TMD (WIIIA VSVVL VAIIA LIIGL SVGK) of Stx2 to generate Stx2-polyK. FAP inserted into the backbone to generate a control. (**b**) Representative confocal image (middle section) of fixed INS832/13 cells expressing FAP-Stx2-polyK incubated with β GREEN-np (n = 3 independent experiments). Scale bar, 5µm. (**c**) Spatial profile of fluorescence intensity of FAP-Stx2-polyK along the line imposed on the image in (**b**) showing that FAP-Stx2-polyK targets to the plasma membrane.

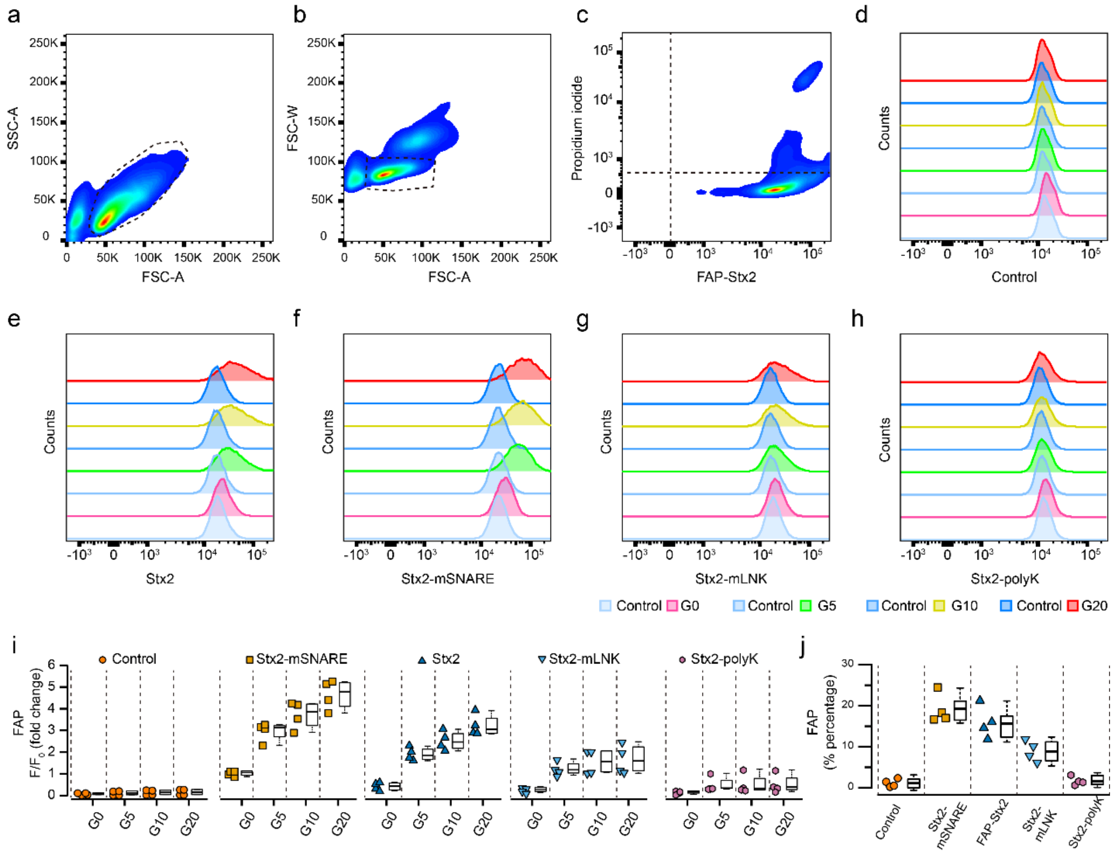

Supplementary Figure 6. Quantification of the flip-out capability of Stx2 chimeras. (**a-c**) Quantification of the flip-out capability of Stx2 chimeras by flow cytometric analysis in INS832/13 cells. Gates were used to exclude non-cell events (gate in **a**), cell doublets and aggregation (gate in **b**). Cells whose membrane permeability were altered and dead cells were excluded using propidium iodide (PI) staining (gate in **c**). (**d-h**) Half-offset histograms show the cell population at resting state (G0), 5min (G5), 10min (G10) and 20min (G20) after glucose stimulation, ~ 2 ×10^4 cells were analyzed for each condition. (**i**) Normalized flip-out capability of chimeras during glucose stimulation (G0 to G20). The transmembrane domain of Stx2 is essential for its flip-out capability. The flip out capability were quantified by $\Delta F/F_0$. (**j**) Flip-out FAP-Stx2s on the cell surface at resting state quantified by $(F_0\text{-}F_{bg})/(F_{max}\text{-}F_{bg})$ (F_{bg} indicates the background intensity). Center lines within the box plots indicate median values, box the interquartile range and whiskers the min/max (n = 4 independent experiments).

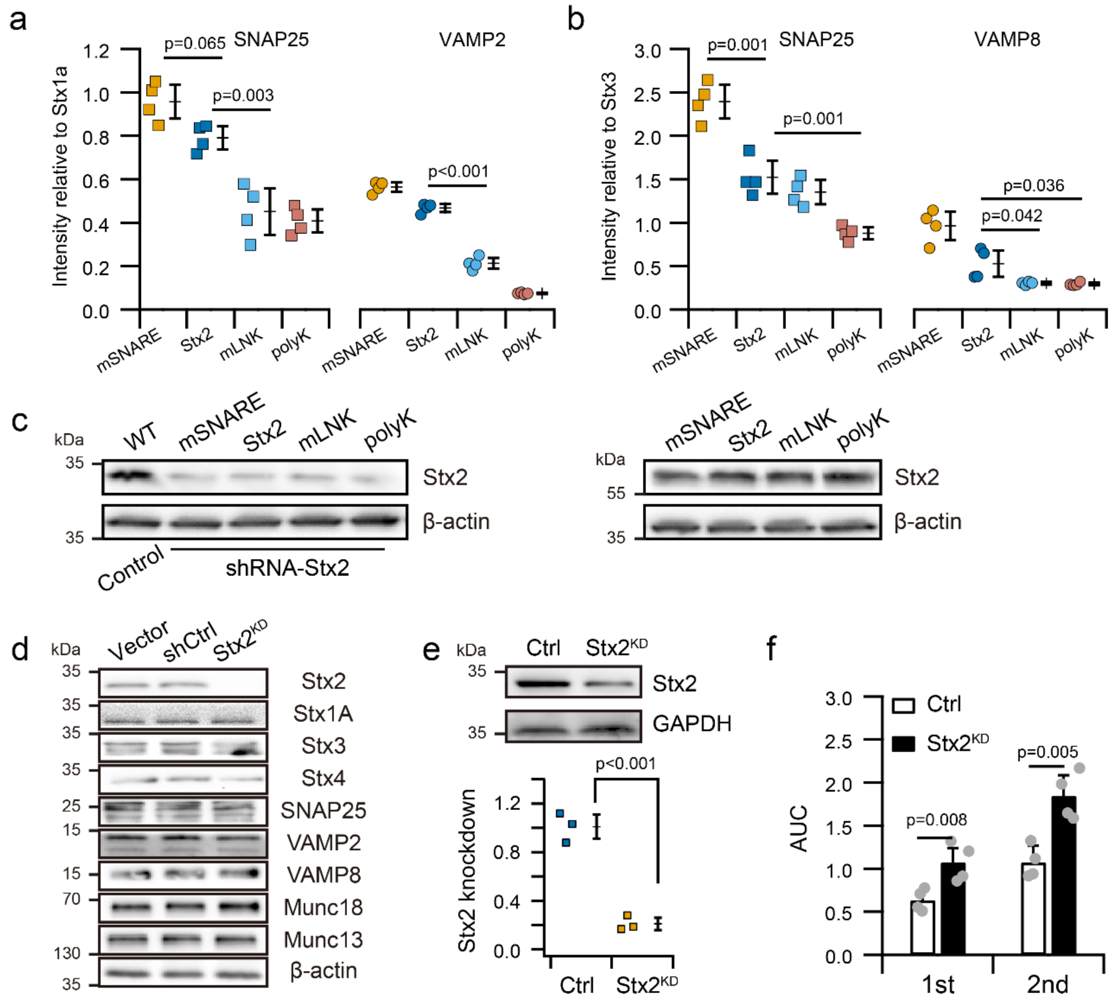

Supplementary Figure 7. Modulating Stx2 flipping efficiency regulates the exocytosis fusion machinery. Analysis of Stx1a (**a**) and Stx3 (**b**) exocytotic SNARE complex formation in human islets infected with the indicated Stx2 chimeras after glucose (16.7 mM) stimulation. Data are presented as mean ± S.D., unpaired Student's *t*-test, from n = 4 independent experiments. (**c**) Western blot analysis of the endogenous Stx2 (*left*) and its mutants' (*right*) expression level using antibody against Stx2 as control for the GSIS and IP assay corresponding to Fig. 6. (**d**) Western blotting of untreated control (vector), non-specific negative control and shRNA-Stx2 treated human islets indicated that other exocytotic syntaxins, SNAP25, VAMPs, and SM and Munc13 proteins were not altered by Stx2 knockdown. The negative control was designed to have no known target to rule out non-specific effects in RNAi experiment, while the untreated control was designed using the blank vector that both negative control and shRNA-Stx2 cloned into to determine the level of cell viability (n=3 independent experiments). (**e**) Ad-Stx2-shRNA knockdown of Stx2 in rat islets, referring to Fig. 6h. Rat islets extracts were prepared from wild type islets infected with Ad-scramble-shRNA (Ctrl) or Ad-Stx2-shRNA, followed by western blotting with the indicated Abs. Data are from three independent experiments presented as mean values ± S.D., unpaired two-tailed Student's *t*-test (**f**) AUC analysis of first and second phase secretion in T2D GK rat islets

corresponding to Fig. 6h. Data are presented as mean values + S.D., unpaired two-tailed Student's *t*-test, n = 4 independent experiments.

References

1. Marchetti P, Bugliani M, De Tata V, Suleiman M, Marselli L. Pancreatic Beta Cell Identity in Humans and the Role of Type 2 Diabetes. *Front Cell Dev Biol* **5**, 55 (2017).
