# Supplementary material for: Plasma membrane flipping of Syntaxin-2 regulates its inhibitory action on insulin granule exocytosis
Source: Nat Commun. 2022 Oct 31;13:6512. doi: 10.1038/s41467-022-33986-3 (PMC9622911; doi:10.1038/s41467-022-33986-3)
Supplement: Supplementary file 3 — Reporting Summary [file 41467_2022_33986_MOESM3_ESM.pdf]

nature portfolio

Corresponding author(s): Herbert Y. Gaisano

Last updated by author(s): Aug 28, 2022

Reporting Summary

Nature Portfolio wishes to improve the reproducibility of the work that we publish. This form provides structure for consistency and transparency in reporting. For further information on Nature Portfolio policies, see our Editorial Policies and the Editorial Policy Checklist.

Statistics

For all statistical analyses, confirm that the following items are present in the figure legend, table legend, main text, or Methods section.

n/a	Confirmed	
	x	The exact sample size (*n*) for each experimental group/condition, given as a discrete number and unit of measurement
	x	A statement on whether measurements were taken from distinct samples or whether the same sample was measured repeatedly
	x	The statistical test(s) used AND whether they are one- or two-sided<br>*Only common tests should be described solely by name; describe more complex techniques in the Methods section.*
x		A description of all covariates tested
x		A description of any assumptions or corrections, such as tests of normality and adjustment for multiple comparisons
	x	A full description of the statistical parameters including central tendency (e.g. means) or other basic estimates (e.g. regression coefficient) AND variation (e.g. standard deviation) or associated estimates of uncertainty (e.g. confidence intervals)
	x	For null hypothesis testing, the test statistic (e.g. *F*, *t*, *r*) with confidence intervals, effect sizes, degrees of freedom and *P* value noted<br>*Give P values as exact values whenever suitable.*
x		For Bayesian analysis, information on the choice of priors and Markov chain Monte Carlo settings
x		For hierarchical and complex designs, identification of the appropriate level for tests and full reporting of outcomes
x		Estimates of effect sizes (e.g. Cohen's *d*, Pearson's *r*), indicating how they were calculated

Our web collection on statistics for biologists contains articles on many of the points above.

Software and code

Policy information about availability of computer code

Data collection	Leica LAS, Lightning Module, Navigator 3.7.2;<br>Nikon NIS 5.2;<br>BD FACSDiva™ v8.0.1;<br>BMG PHERAstar FS;
Data analysis	Igor Pro 6.11;<br>tracking algorithm, u-track 2.0, Matlab R2019b (MathWorks);<br>SnapGene Viewer 6.1.1;<br>FlowJo v10;<br>Fiji, win 64 bit v1.53;<br>Microsoft Excel 2016;<br>Adobe illustrator CS6;

For manuscripts utilizing custom algorithms or software that are central to the research but not yet described in published literature, software must be made available to editors and reviewers. We strongly encourage code deposition in a community repository (e.g. GitHub). See the Nature Portfolio guidelines for submitting code & software for further information.

Data

Policy information about availability of data

All manuscripts must include a data availability statement. This statement should provide the following information, where applicable:

- Accession codes, unique identifiers, or web links for publicly available datasets
- A description of any restrictions on data availability
- For clinical datasets or third party data, please ensure that the statement adheres to our policy

The authors declare that all data supporting the findings of this study are available within the paper and/or the Supplementary Information/Source data file.

Human research participants

Policy information about studies involving human research participants and Sex and Gender in Research.

Reporting on sex and gender	No sample size calculation was performed, and sample size was based on previous experience with single cells and human islets (PMID: 32051343, PMID: 28115395, PMID: 24835618, PMID: 28481223) as well as experience and common standards in similar field for calculating statistical significance and also dependent on donor availability.
Population characteristics	Human islets were from 21 non-diabetic donors (14 male and 7 female, age 19.8~37.2) and human pancreata were from 8 non-diabetic donors (Trillium Gift of Life Network, Canada) (4 male and 4 female, age 17.1~29.5).
Recruitment	Human pancreatic islets were purchased from the IsletCore, University of Alberta. Cadaveric donor pancreata deemed unsuitable for transplantation were obtained from Trillium Gift of Life Network, Canada. No selection criteria were applied.
Ethics oversight	Human pancreas obtained from normal portions of pancreatic operations. Human pancreatic islets were purchased from the IsletCore, University of Alberta, which were from institutional review board-approved donors with preoperative written informed consent for research by donors themselves and their family.<br><br>All animal procedures and use of human pancreas were carried out in accordance with ethical guidelines of the University of Toronto's Animal Care Committee and Research Ethics Board of the University Health Network, Toronto, ON, Canada, and with approved IRBs.

Note that full information on the approval of the study protocol must also be provided in the manuscript.

Field-specific reporting

Please select the one below that is the best fit for your research. If you are not sure, read the appropriate sections before making your selection.

☒ Life sciences ☐ Behavioural & social sciences ☐ Ecological, evolutionary & environmental sciences

For a reference copy of the document with all sections, see nature.com/documents/nr-reporting-summary-flat.pdf

Life sciences study design

All studies must disclose on these points even when the disclosure is negative.

Sample size	No sample-size calculation was performed. Sample sizes were decided based on previous publications (PMID: 28115395, PMID: 15339904, PMID: 32051343, PMID: 28031464, PMID: 32312960), experience and common standards in similar field for calculating statistical significance and also dependent on donor availability, a minimum of three independent experiments were carried out. Sample numbers are indicated in the figure legends. Multiple tests and analyses were performed as described in the manuscript to ensure the samples are representative and results are conclusive.
Data exclusions	No data were excluded.
Replication	The number of replicates for each specific experiment is indicated throughout the manuscript text, figure legends and methods. All attempts of replication were successful.
Randomization	Not relevant. For each group, islet samples from each donor were equally divided onto several coverslips that were treated differently only during data acquisition. All experiments were performed with appropriate negative and positive controls in keeping with the standards of the field.
Blinding	Data files from individual donors were processed together and without knowledge of the treatment group (except for donor information, which was known on arrival of islet samples).

Reporting for specific materials, systems and methods

We require information from authors about some types of materials, experimental systems and methods used in many studies. Here, indicate whether each material, system or method listed is relevant to your study. If you are not sure if a list item applies to your research, read the appropriate section before selecting a response.

Materials & experimental systems

n/a	Involved in the study
☐	☒ Antibodies
☐	☒ Eukaryotic cell lines
☒	☐ Palaeontology and archaeology
☒	☐ Animals and other organisms
☒	☐ Clinical data
☒	☐ Dual use research of concern

Methods

n/a	Involved in the study
☒	☐ ChIP-seq
☐	☒ Flow cytometry
☒	☐ MRI-based neuroimaging

Antibodies

Antibodies used

The separated proteins detected by primary antibodies (SNAP-25, SySy, #111 002, 1:1000; Stx1A, Sigma, # SAB5500181, 1:1000, SySy, #110 302, 1:1000; VAMP2, R&D, #AF5136, 1:500; Stx2, Abcam, #ab12369, 1:500; SySy, #110 123, 1:500; Stx3, SySy, #110 033, 1:1000; VAMP8, SySy, #104 303, 1:500; β-actin, Abcam, #ab8227, 1:10,000) and horseradish peroxidase conjugated secondary antibody (Jackson, AffiniPure Goat Anti-Rabbit IgG (H+L), #111-005-144; AffiniPure Goat Anti-Mouse IgG (H+L), #115-005-003). The secondary antibodies were incubated for 1h at a concentration of 1:2500. For IP experiments, native IgG-specified secondary antibodies (Abcam) were used to avoid the detection of the denatured primary antibody heavy and light chains at 1:1000 dilution for 1h at room temperature.

Primary antibody against ANTI-HPI2, CLONE HIC1-2B4 (Sigma-Aldrich, MABS1999-100UG, 1:500) and ANTI-HPA3, CLONE HIC3-2D12 (Sigma-Aldrich, MABS1998, 1:500) were obtained from Millipore Sigma. Secondary antibody, Goat Anti-Mouse IgM mu chain (DyLight® 550) (Abcam, ab98675, 1:1500) and Goat Anti-Mouse IgG2b heavy chain (PE/Cy7 ®) (ab130790, 1:1500) were purchased from Abcam.

Validation

All antibodies commercially available were validated by the manufacturer and published research articles.

SNAP-25, blocking buffer:10% Bovine Serum Albumin; Immunogen: Synthetic peptide corresponding to AA 192 to 206 from human SNAP25; Reactivity:human, rat, mouse; Specificity Statement: The antibody reacts specifically against human and rat, both of which validated by western blots. Selected Validation Data available on supplier website and our previous publication, PMID: 32051343 and PMID: 28115395.

Stx1A, blocking buffer:10% Bovine Serum Albumin; Immunogen: Synthetic peptide corresponding to AA 172 to 189 from rat Syntaxin1A; Reactivity:human, rat, mouse; Specificity Statement: The antibody reacts specifically against human and rat, both of which validated by IP and western blots. Validation Data available on supplier website and our previous publication, PMID: 32051343 and PMID: 28115395.

VAMP2, blocking buffer:10% Bovine Serum Albumin; Immunogen: E. coli-derived recombinant human VAMP-2; Reactivity:human, rat, mouse; Specificity Statement: The antibody reacts specifically against human and rat, which validated by Immunohistochemistry and western blots. Validation Data available on supplier website and our previous publication, PMID: 32051343 and PMID: 28115395.https://www.rndsystems.com/products/human-mouse-rat-vamp-2-antibody_af5136

Stx2 (Syntaxin2), blocking buffer:10% Bovine Serum Albumin; Immunogen: Recombinant protein corresponding to AA 1 to 265 from rat Syntaxin2; Reactivity:human, rat, mouse, hamster.; Specificity Statement: The antibody reacts specifically against human and rat, hamster. The reactivity to human and rat validated by western blots. Validation Data available on our previous publication, PMID: 32051343 and other's publication, PMID: 32994278 and PMID: 31297933.

Stx3 (Syntaxin3), blocking buffer:10% Bovine Serum Albumin; Immunogen: Recombinant protein corresponding to AA 1 to 260 from rat Syntaxin3; Reactivity:human, rat, mouse, hamster; Specificity Statement: The antibody reacts specifically against human and rat, both of which validated by western blots. Validation Data available on supplier website and our previous publication, PMID: 32051343 and other's publication, PMID: 35045291.

VAMP8, blocking buffer:10% Bovine Serum Albumin; Immunogen: Recombinant protein corresponding to AA 1 to 75 from rat VAMP8; Reactivity:human, rat, mouse; Specificity Statement: The antibody reacts specifically against human and rat, which validated by western blots. Validation Data available on supplier website and our previous publication, PMID: 32051343, PMID: 28115395 as well as other's publication, .PMID: 35045291.

β-actin, used as loading control, blocking buffer:10% Bovine Serum Albumin; Immunogen: Synthetic peptide. This information is proprietary to Abcam and/or its suppliers. Specificity Statement: The antibody reacts specifically against human, mouse and rat, which validated by western blots. This loading control is widely used by more than 3000 publications. Validation Data available on supplier website and our previous publication, PMID: 32051343, PMID: 28115395 as well as other's publication, .PMID: 33262832, 32963176.

Eukaryotic cell lines

Policy information about cell lines and Sex and Gender in Research

Cell line source(s)

HEK293A and HEK293T cells were obtained from ATCC, Canada.
INS832/13 cells were obtained from C. Newgard, Duke University, Durham, NC., which originally from Sigma.

Authentication	All cell lines were either purchased directly from the above corporations or directly mailed from the gifting labs, but not independently authenticate them.
Mycoplasma contamination	All cell lines were bi-monthly tested for mycoplasma contamination utilizing PCR detection kit and confirmed negative.
Commonly misidentified lines (See ICLAC register)	None.

Flow Cytometry

Plots

Confirm that:

- [x] The axis labels state the marker and fluorochrome used (e.g. CD4-FITC).
- [x] The axis scales are clearly visible. Include numbers along axes only for bottom left plot of group (a 'group' is an analysis of identical markers).
- [x] All plots are contour plots with outliers or pseudocolor plots.
- [x] A numerical value for number of cells or percentage (with statistics) is provided.

Methodology

Sample preparation	Human islets were dispersed into single cells for flow cytometry analysis; this is by digestion in cell dissociation buffer at 37°C with shaking dispersal every 1-2 min. Dispersed cells were cultured in DMEM (low-glucose, Gibco) with L-glutamine, supplemented with 10 % fetal bovine serum (FBS), 110 mg/L sodium pyruvate and 100 U/mL penicillin/streptomycin at 37°C and 5% CO_2. Additionally, to prevent clogging, dispersed cells were filtered by nylon mesh with a pore size of 40 µm (BD Falcon) prior to loading into flow cytometry instrument.
Instrument	Cells were analyzed and sorted with BD Fortessa X20 (Becton-Dickenson).
Software	FlowJo analysis software (Version 10.1, TreeStar Inc., San Carlos, CA, USA) was used for analysis of all flow cytometry data. Gates defining positive and negative populations for specific markers were described in supplementary information.
Cell population abundance	The abundance of relevant populations is quantified and presented throughout the manuscript. Re-sorting of gated collected fractions was used during the initial FACS quality control to evaluate protocols.
Gating strategy	Propidium iodide (ThermoFisher, P1304MP) staining was used to label dead cells for exclusion and cell doublets were excluded by pulse width measurement of forward scatter (FSC). Rainbow Fluorescent Particles (RFP-30-5A) was used to standardize FACS. A figure exemplifying the gating strategy is provided in Supplementary Information figure S6.

- [x] Tick this box to confirm that a figure exemplifying the gating strategy is provided in the Supplementary Information.
